# Five-year survival outcomes of intensity-modulated radiotherapy with simultaneous integrated boost (IMRT-SIB) using forward IMRT or Tomotherapy for breast cancer

**DOI:** 10.1038/s41598-020-61403-6

**Published:** 2020-03-09

**Authors:** Hsin-Hua Lee, Chien-Hung Chen, Kuei-Hau Luo, Hung-Yi Chuang, Chih-Jen Huang, Yuan-Kai Cheng, Frank Chen, Shih-Hsun Kuo, Ming-Yii Huang

**Affiliations:** 1 Ph.D. Program in Environmental and Occupational Medicine, Kaohsiung Medical University and National Health Research Institutes, Kaohsiung, Taiwan; 2 Department of Radiation Oncology, Kaohsiung Medical University Hospital, Kaohsiung, Taiwan; 3 Department of Radiation Oncology, Kaohsiung Municipal Ta-Tung Hospital, Kaohsiung, Taiwan; 4 Graduate Institute of Medicine, College of Medicine, Kaohsiung Medical University, Kaohsiung, Taiwan; 5 Department of Occupational and Environmental Medicine, Kaohsiung Medical University Hospital, Kaohsiung Medical University, Kaohsiung, Taiwan; 6 Department of Radiation Oncology, Faculty of Medicine, College of Medicine, Kaohsiung Medical University, Kaohsiung, Taiwan; 7 Center for Biomarkers and Biotech Drugs, Kaohsiung Medical University, Kaohsiung, Taiwan; 8 Center for Cancer Research, Kaohsiung Medical University, Kaohsiung, Taiwan

**Keywords:** Breast cancer, Breast cancer

## Abstract

Intensity-modulated radiotherapy with simultaneous integrated boost (IMRT-SIB) reduces overall treatment duration and results in less radiotherapy (RT)-induced dermatitis. However, the use of traditional sequential approach or IMRT-SIB is still under debate since there is not enough evidence of long-term clinical outcomes. The present study investigated 216 patients who underwent breast conserving surgery (BCS) between 2010 and 2013. The median age was 51 years (range, 21–81 years). All patients received IMRT-SIB, 50.4 Gy at 1.8 Gy per fraction to the whole breast and 60.2 Gy at 2.15 Gy per fraction to the tumor bed by integral boost. Among 216 patients, 175 patients received post-operative RT with forward IMRT and 41 patients had Tomotherapy. The median follow-up was 6.4 years. Forty patients (97.6%) in the Tomotherapy arm and 147 patients (84%) in the IMRT arm developed grade 0–1 skin toxicity (*P* = 0.021). For the entire cohort, the 5-year and 7-year overall survival (OS) rates were 94.4% and 93.1% respectively. The 7-year distant metastasis-free survival rates were 100% vs 89.1% in the Tomotherapy and IMRT arm respectively (*P* = 0.028). In conclusion, Tomotherapy improved acute skin toxicity compared with forward IMRT-SIB. Chronic skin complication was 1.9%. IMRT-SIB resulted in good long-term survival.

## Introduction

Breast cancer is the most commonly diagnosed cancer and the leading cause of cancer death in women globally^1^. The long-term survival of women with early breast cancer who were treated with breast-conserving surgery (BCS) and postoperative radiotherapy (RT) was the same when matched with the rate among women who underwent radical mastectomy^2,3^. The 20-year overall (OS) and breast-cancer-specific survival (CSS) rates were similar in the two groups^3^. Furthermore, additional RT boost to the surgical bed is found to improve 10-year local tumor control^4^. The most common adjuvant RT after BCS is conventionally administered in a 7–8 week period, with doses of 1.8–2.0 Gy per fraction to a total dose of approximately 50 Gy followed by a sequential boost irradiation of 10–16 Gy to the tumor bed.

Since the last decade, there has been an emerging role of hypofractionated RT for patients with breast cancer after BCS^5,6^. Hypofractionation uses a lower total dose and reduces acute toxicity compared with conventional schedules^7–9^. With technology advancement, intensity-modulated radiotherapy (IMRT) integrates the boost concept in the daily radiation sessions by increasing the dose per fraction within the boost volume^10^. This is the so-called IMRT with simultaneous integrated boost (SIB). SIB with more homogeneous dose distributions has been implemented in clinical routines nowadays^11^. The rationale of IMRT-SIB is the reduction of overall treatment duration in view of less RT-induced side effect; nonetheless, the use of sequential or SIB in patients treated with hypofractionated RT is still under debate since there is not enough evidence of long-term survival.

To the best of our knowledge, no evaluation of long-term survival between forward and inverse IMRT-SIB has been reported. We previously demonstrated that conventional RT with sequential boost for post-operative treatment of breast cancer resulted in more severe acute dermatological toxicity compared to IMRT-SIB^12^. The endpoints of this study are the long-term survival rates after forward IMRT-SIB or inverse IMRT-SIB performed by Tomotherapy.

## Methods

### Patients

This retrospective study comprised 216 consecutive female patients who were diagnosed with pathologically-proven breast cancer between March 2010 and June 2013. The exclusion criteria included excluding patients with synchronous bilateral breast cancer, or a history of previous irradiation to the thorax, or neo-adjuvant chemotherapy. We staged all patients by the 2010 TNM classification system (AJCC 7)^13^ and collected the data regarding post-RT acute and chronic skin reaction, date of diagnosis, adjuvant chemotherapy, hormonal treatment, RT treatment planning, pathological reports including primary surgery, hormonal receptor status and Human epidermal growth factor receptor 2 (Her2) over-expression status. The study was approved by the Ethical and Research Committee in the university hospital (KMUHIRB-E(I)- 20190053) and it was conducted under compliance of the Institutional Review Board regulations in accordance with the Helsinki Declaration of 1975 as revised in 1983. All the patients provided written informed consent for treatment prior to surgery and RT. All data approved by the Ethical committee were anonymized and de-identified for analysis.

### Radiotherapy

All patients in this study were treated with a plan that integrated both breast and boost beams individually designed for herself. The fractionation schemes were 60.2 Gy to the tumor bed and 50.4 Gy to the whole breast. Such scheme was biologically equivalent to the traditional sequential boost-technique consisting of 50 Gy to the whole breast followed by a boost irradiation of 12 Gy in 6 fractions, using an alpha/beta ratio of 4 Gy for tumor response^14^. We recorded acute and chronic skin reactions during routine follow-up in accordance with the Common Terminology Criteria of Adverse Events version 4.03 (CTCAE v4.03).

After all organs at risks (OAR) and region of interest were contoured manually from axial-computed tomography (CT) images^12^, we utilized the Hi-Art helical Tomotherapy, version 2.2.4.1 (TomoTherapy, Inc., Madison, WI) unit or Eclipse, version 8.6 (Varian medical Systems Inc., Palo Alto, USA) to make IMRT-SIB treatment plans. IMRT were planned forwardly or inversely. We covered the PTV with the 95% iso-dose line, and minimized the volumes receiving higher than 110% of the dose prescribed to the PTV. Dose volume constraints for OAR were: whole lung V20Gy <20% and heart V25Gy <10%. Tomotherapy combines a rotational inverse IMRT with a translational movement of the couch^15,16^. Volumetric arc planning was not used.

### Systemic therapy

The patients with either node-positive disease or high risk node-negative tumors received adjuvant chemotherapy after BCS. Based on tumor size, grading, hormonal receptor status and age, the risk was determined individually at the discretion of the physician. The chemotherapy regimen, adjuvant hormonal therapy and the use of Trastuzumab were detailed in our previous report^12^.

### Statistical analysis

Firstly, we used Pearson’s chi-square test for categorical variables or Student’s *t*-test for continuous variables to compare the demographic characteristics and clinical variables between Tomotherapy and IMRT. Then we performed multiple logistic regressions to compute the adjusted ORs and 95% CIs with SPSS software package, version 20.0 for Windows (SPSS, Chicago, IL, USA). P <0.05 was considered statistically significant.

## Results

The median age of this retrospective cohort was 51 years (range, 21–81 years). Table 1 summarizes the clinical characteristics of the 216 patients, divided by planning method into IMRT and Tomotherapy. The median follow-up was 6.4 years (range: 476 days – 2868 days). Forty-one patients (19%) received IMRT-SIB via Tomotherapy and 175 patients (81%) underwent IMRT-SIB. No significant difference was observed in terms of age, laterality, pathological tumor or nodal classification, pathological stage, hormonal receptors, the addition of chemotherapy or hormonal therapy, Her2 over-expression, surgical margin, V20 for whole lung, or chronic dermatological complications. Ductal carcinoma was found in 70.7% and 85.1% of Tomotherapy and IMRT arms respectively (*P* = 0.04). Both arms had acceptable V25 to the heart, yet the median V25 to the heart was smaller in the IMRT arm (*P* = 0.004).

**Table 1:** Patient and treatment characteristics.

	Entire cohort (n = 216)	IMRT (n = 175)	Tomotherapy (n = 41)	*P*-value
Age (year)				0.118
Mean (year)	51.7	51.2	54.0	
Median	51	50	53	
Range	21–81	21–81	39–78	
Age (year)				0.100
≤50	104 (48.1%)	88 (50.6%)	16 (38.1%)	
>50	112 (51.9%)	86 (49.4%)	26 (61.9%)	
Laterality				0.864
Left	96 (44.4%)	78 (44.8%)	18 (42.9%)	
Right	120 (55.6%)	96 (55.2%)	24 (57.1%)	
Pathology				0.040
Ductal carcinoma	178 (82.4%)	148 (85.1%)	30 (70.7%)	
Others	38 (17.6%)	26 (14.9%)	12 (29.3)	
Pathological Tumor classification				0.295
0–1	170 (78.7%)	135 (77.1%)	35 (85.4%)	
2–4	46 (21.3%)	40 (22.9%)	6 (14.6%)	
Pathological Nodal classification				0.258
N0–N1	192 (74.1%)	159 (94.6%)	33 (89.2%)	
N2–N3	13 (25.9%)	9 (5.4%)	4 (10.8%)	
Pathological stage				0.055
0	10 (4.6%)	6 (3.4%)	4 (9.5%)	
1	139 (64.4%)	117 (67.2%)	22 (52.4%)	
2	51 (23.6%)	41 (23.6%)	10 (23.8%)	
3	14 (6.5%)	8 (4.6%)	6 (14.3%)	
4	2 (0.9%)	2 (1.1%)	0 (0.0%)	
Estrogen receptor status				0.080
Negative	37 (17.1%)	32 (18.4%)	5 (11.9%)	
Positive	178 (82.4%)	142 (81.6%)	36 (85.7%)	
Uncertain	1 (0.5%)	0 (0.0%)	1 (2.6%)	
Progesterone receptor status				0.119
Negative	62 (28.7%)	51 (29.3%)	11 (26.2%)	
Positive	153 (7.8%)	123 (70.7%)	30 (71.4%)	
Uncertain	1 (0.5%)	0 (0.0%)	1 (2.4%)	
Her2/neu overexpression				0.194
Negative	168 (77.8%)	131 (75.3%)	37 (88.1%)	
Positive	47 (21.8%)	42 (24.1%)	5 (11.9%)	
Uncertain	1 (0.5%)	1 (0.6%)	0 (0.0%)	
Chemotherapy				0.769
No	19 (8.8%)	15 (8.6%)	4 (9.5%)	
Yes	197 (91.2%)	159 (91.4%)	38 (90.5%)	
Hormone therapy				0.654
No	38 (17.6%)	32 (18.4%)	6 (14.3%)	
Yes	178 (82.4%)	142 (81.6%)	36 (85.7%)	
Tumor grading				0.493
1	38 (17.6%)	29 (16.7%)	9 (21.4%)	
2	112 (51.9%)	88 (50.6%)	24 (57.1%)	
3	54 (25.0%)	46 (26.4%)	8 (19.0%)	
Uncertain	12 (5.6%)	11 (6.3%)	1 (2.4%)	
Surgical margin				>0.999
Negative	203 (94.9%)	164 (94.8%)	39 (95.1%)	
Microscopic	11 (5.1%)	9 (5.2%)	2 (4.9%)	
V20 (whole lung)	8.6 (2.7)	8.7 (2.4)	8.1 (3.8)	0.352
V25 Gy (heart)	3.5 (4.5)	3.1 (4.4)	5.3 (4.7)	0.004
Mean heart (Gy)	4.9 (4.2)	3.6 (3.1)	10.7 (3.4)	<0.001
V20 (whole lung)				0.334
<20	214 (99.1%)	174 (99.4%)	40 (97.6%)	
≧20	2 (0.9%)	1 (0.6%)	1 (3.4%)	
Acute Skin toxicity				0.021
0–1	187 (86.6%)	147 (84.0%)	40 (97.6%)	
2–3	29 (13.4%)	28 (16.0%)	1 (2.4%)	
Chronic Skin effect				0.572
No	212 (98.1%)	172 (98.3%)	40 (97.6%)	
Yes	4 (1.9%)	3 (1.7%)	1 (2.4%)	

Abbreviations: IMRT: intensity modulated radiotherapy.

### Acute and chronic skin toxicity

For the entire cohort, 187 patients (86.6%) had grade 0–1 acute RT-induced dermatitis. Twenty-three patients (13.1%) in the IMRT-SIB arm and 10 patients (24.4%) in the Tomotherapy arm had grade 0 dermatitis. Most of the patients who developed RT-induced dermatitis had acute grade 1 erythema during RT. In the majority of cases, 124 patients (70.9%) in the IMRT-SIB arm and 30 patients (73.2%) in the Tomotherapy arm had grade 1 dermatitis. Among 216 patients, only one patient (0.6%) had grade 3 acute toxicity. This patient was in the IMRT group. There was no grade 4 toxicity. All patients in the Tomotherapy group experienced grade 0–2 toxicity, with no cases ≥ grade 3. Forty patients (97.6%) in the Tomotherapy arm and 147 patients (84%) in the IMRT arm developed grade 0–1 skin toxicity (*P* = 0.021). Less patients suffered from grade 2–3 RT-induced dermatitis (*P* = 0.021) in the Tomotherapy arm. Twenty-seven patients (15.4%) in the IMRT-SIB arm and 1 patient (2.4%) in the Tomotherapy arm developed grade 2 dermatitis.

Chronic grade 1 skin toxicity was recorded in four patients (1.9%). They had grade 1 late effect such as induration or fibrosis without telangiectasia. One patients in the Tomotherapy arm and 3 patients in the IMRT arm developed late grade 1 skin toxicity (*P* = 0.572).

### Survival

**Figure 1:**
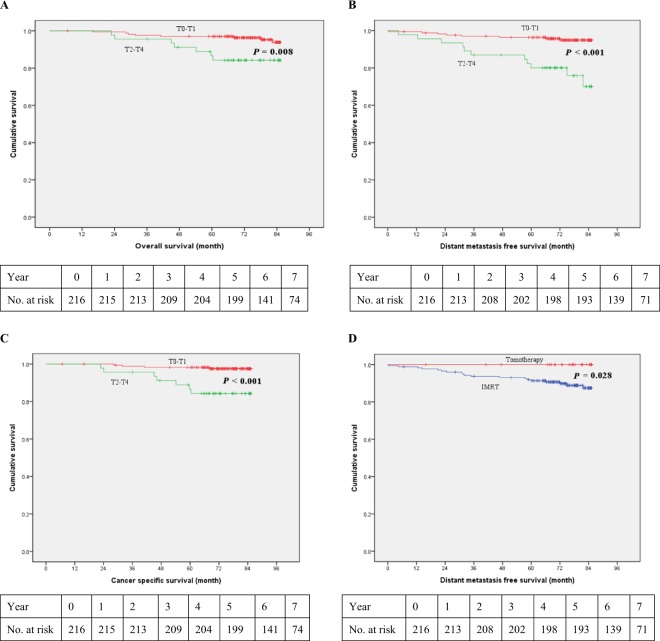
(**A**) Kaplan*-*Meier curves of overall survival according to tumor size (Log Rank test, *P* = 0.008). (**B**) Kaplan*-*Meier curves of distant metastasis-free survival according to tumor size (Log Rank test, *P* < 0.001). (**C**) Kaplan*-*Meier curves of cancer-specific survival according to tumor size (Log Rank test, *P* < 0.001). (**D**) Kaplan*-*Meier curves of distant metastasis-free survival according to radiotherapy modality (Log Rank test, *P* = 0.028).

Table 2 shows the survival rates and the comparison between IMRT and Tomotherapy. For the entire cohort, the 5-year and 7-year OS rates were 94.4% and 93.1% respectively. Figures 1A–C are the Kaplan-Meier curves of OS, cancer specific survival (CSS) and distant metastasis-free survival (DMFS) divided by T classification (T0–1 versus T2–4). In Table 3, univariate analysis suggested that pT0–1, pN0–1, pathological stage 0–1, ER (+), PR (+), and the use of hormone therapy were favorable prognostic factors for longer OS. After controlling for significant covariables in a multivariable model, pathological stage and ER (+) were associated with improved OS.

**Table 2:** Survival outcomes.

	Entire cohort (n = 216)		IMRT (n = 175)		Tomotherapy (n = 41)		*P-*value	
	5 yr (%)	7 yr (%)	5 yr (%)	7 yr (%)	5 yr (%)	7 yr (%)	5 yr	7 yr
Overall survival	94.4	93.1	94.3	92.6	95.1	95.1	0.840	0.528
Local regional disease-free survival	94.9	94.0	94.9	93.7	95.1	95.1	0.959	0.707
Distant metastasis-free survival	93.1	91.2	91.4	89.1	100%	100%	0.058	**0.028**
Cancer-specific survival	95.4	94.9	94.9	94.3	97.6%	97.6	0.935	0.397

Abbreviations: IMRT: intensity-modulated radiotherapy.

**Table 3:** Univariate and Multivariate Cox regression analyses of co-variables associated with overall survival.

	Univariate analysis	P-value	Multivariate analysis	P-value
Age (>50 vs ≤50)	1.021 (0.370–2.819)	0.968	0.909 (0.325–2.542)	0.856
Treatment (Tomotherapy vs IMRT)	0.622 (0.140–2.758)	0.532	0.616 (0.136–2.793)	0.530
Pathological Tumor classification (T2–4 vs T0–1)	3.591 (1.300–9.922)	0.014		
Pathological Nodal classification (N2–3 vs N0–1)	4.920 (1.366–17.715)	0.015		
Pathological stage (2–4 vs 0–1)	3.608 (1.283–10.145)	0.015	**3.223** (**1.131–9.182)**	**0.028**
Pathology (Ductal carcinoma vs others)	27.487 (0.097–7805.220)	0.250		
Estrogen receptor status (Positive vs Negative)	0.222 (0.080–0.611)	0.004	**0.259** (**0.093–0.725)**	**0.010**
Progesterone receptor status (Positive vs Negative)	0.338 (0.122–0.931)	0.036		
Her-2 overexpression (Positive vs Negative)	1.445 (0.453–4.608)	0.534		
Chemotherapy (Yes vs No)	22.966 (0.007–71847.098)	0.445		
Hormone therapy (Yes vs No)	0.227 (0.082–0.627)	0.004		
Tumor grading (3 vs 1–2)	1.667 (0.557–4.992)	0.361		
Margin	1.549 (0.203–11.817)	0.673		
Acute skin toxicity (2–3 vs 0–1)	0.508 (0.066–3.877)	0.513		

Abbreviations: IMRT: intensity-modulated radiotherapy.

Since the 7-year (100% vs 89.1%, *P* = 0.028, Log rank; Fig. 1D) DMFS in Tomotherapy was significantly longer than that in the IMRT arm, we performed Cox regression as shown in Table 4, illustrating that the pathological stage 0–1 (hazard ratio [HR], 6.974; 95% CI, 2.471 to 19.681; *P* < 0.001) was an independent favorable prognostic factor for DMFS in multivariate analysis. Tomotherapy did not confer a significant DMFS benefit in multivariate analysis (*P* = 0.971).

**Table 4:** Univariate and Multivariate Cox regression analyses of co-variables associated with distant metastasis-free survival.

	Univariate analysis	P-value	Multivariate analysis	P-value
Age (>50 vs ≤50)	1.217 (0.489–3.026)	0.673	1.059 (0.423–2.650)	0.902
Treatment (Tomotherapy vs IMRT)	0.035 (0.000–4.311)	0.172	0.000	0.971
Pathological Tumor classification (T2–4 vs T0–1)	5.673 (2.278–14.128)	<0.001		
Pathological Nodal classification (N2–3 vs N0–1)	5.052 (1.672–15.271)	0.004		
Pathological stage (2–4 vs 0–1)	6.844 (2.464–19.011)	<0.001	6.974 (2.471–19.681)	<0.001
Pathology (Ductal carcinoma vs others)	1.183 (0.345–4.061)	0.789		
Estrogen receptor status (Positive vs Negative)	0.414 (0.157–1.091)	0.074		
Progesterone receptor status (Positive vs Negative)	0.419 (0.170–1.033)	0.059	0.563 (0.227–1.399)	0.216
Her-2 overexpression (Positive vs Negative)	2.332 (0.904–6.017)	0.080		
Chemotherapy (Yes vs No)	23.049 (0.020–26642.710)	0.383		
Hormone therapy (Yes vs No)	0.425 (0.162–1.119)	0.083		
Tumor grading (3 vs 1–2)	1.222 (0.430–3.475)	0.707		
Margin	1.072 (0.392–2.937)	0.892		
Acute skin toxicity (2–3 vs 0–1)	1.374 (0.398–4.742)	0.615		

Abbreviations: IMRT: intensity modulated radiotherapy.

## Discussion

To the best of our knowledge, the present study is the first to evaluate the differences of survival rates longer than five years between forward IMRT-SIB and inverse IMRT-SIB via Tomotherapy. Medical physicists specified beam parameters and manually optimized them in forward-planned IMRT which involves multi-leaf collimators to create a nonuniform fluence. On the other hand, inverse-planned IMRT uses optimization algorithms to create fluence maps and shape dose distributions^17^. Many researchers have discovered the merits of IMRT, SIB or Tomotherapy for lessening skin reaction and have reported safe short-term toxicity profiles^9,18–20^. More than a decade ago, Pignol *et al*. had documented that breast IMRT significantly reduced the occurrence of moist desquamation compared with the traditional wedged technique^21^. More recent methods to optimize the delivery of ionizing radiation have included three-dimensional conventional RT (3D-CRT) incorporating SIB, IMRT-SIB, VMAT-SIB and Tomotherapy^22,23^. IMRT plans reduce the unwanted excessive dose to the breast compared with the conventional photon boost plan, especially for the patient with a deep-seated tumor^24–26^. Increasing relevant evidence has been generated to consider SIB as an alternative to traditional sequential techniques^27^.

Hammer *et al*., reported that when 3D-CRT incorporated SIB, chronic grade 2 fibrosis was observed in 13.4% of 546 patients^28^. De rose *et al*. reported a phase II trial of 787 patients that used VMAT-SIB technique to the whole breast and tumor bed in 15 fractions, for a total dose of 40.5 and 48 Gy^29^. At the end of RT in their study, acute skin toxicity was grade 1 in 51.1% of all patients, and grade 2 in 9.7%. In the present study of IMRT-SIB, 71.3% of all patients had acute grade 1 and 13% had grade 2. At two years of follow-up, De rose *et al*. noted chronic grade 1 in 13.5% of patients. The chronic skin complication rate in our study after a median follow-up of 6.4 years was 1.9%, with no cases ≥grade 2. Milder acute dermatitis was observed in the Tomotherapy arm (*P* = 0.021).

In the aspect of survival, McDonald *et al*. compared IMRT with conventional 3D-CRT. They reported no statistically significant difference in OS, CSS, or recurrence, DMFS, late toxicity, or second malignancies after a median follow-up of 6.3 years^30^. Furthermore, the same team utilized IMRT-SIB, delivering 1.8 Gy to surrounding breast tissue and 2.14 Gy to the surgical bed simultaneously, yielding a breast dose of 45 Gy in 25 fractions and cavity dose of 59.92 Gy in 28 fractions^31^. This is similar to our SIB regimen of 60.2 Gy and 50.4 Gy in 28 fractions.

Until recently, there was no long-term result from prospective randomized trials regarding IMRT-SIB^32^. The present study demonstrated 5-year and 7-year OS, DMFS and CSS from forward IMRT-SIB and inverse IMRT-SIB via Tomotherapy. In the present study, positive ER (HR, 0.259; 95% CI, 0.093 to 0.725; *P* = 0.010) was an independent favorable prognostic factor and having pathological stage 2–4 (HR, 3.223; 95% CI, 1.131 to 9.182; *P* = 0.028) was an independent unfavorable prognostic factor. The mechanism for the significantly longer 7-year DMFS in Tomotherapy arm is unclear (100% vs 89.1%, *P* = 0.028), since there were more infiltrating ductal carcinomas (IDC) in the IMRT arm (85.1% vs 70.7%, *P* = 0.04). Other pathological types included infiltrating lobular carcinoma (ILC) and mucinous carcinomas were more common in the Tomotherapy arm. Chen *et al*. reported that the prognosis of ILC is poorer than that of IDC^33^. They found higher percentages of metastatic lymphadenopathy and distant metastases in ILC. Some studies have reached different conclusions and documented that ILC had better prognosis than IDC^34,35^. One study suggested that ILC had higher risk of metastatic disease^36^. Our multivariable analysis revealed that only pathological stages 0–1 (HR, 6.974; 95% CI, 2.471 to 19.681; *P* < 0.001) was an independent favorable prognostic factor for DMFS (Table 4).

SIB delivers different doses to different target volumes within a single RT fraction^15^. It reduces the overall treatment time and lowers the expense for patients^37^. We believe SIB is more economically efficient in terms of time and money. Tomotherapy appears to improve target coverage while sparing OAR because of its high conformity; when paired with SIB, it maintains the ability to deliver adequate dose coverage^38^. Studies have reported that helical Tomotherapy avoided unnecessary breast overdose while improving ipsilateral lung dosimetry^16,38^; furthermore, static ports of Tomotherapy in TomoDirect were proven to prevent unwanted dosages to the surrounding normal tissues^39^. Tomotherapy significantly reduced cardiac doses and slightly increases in dosage to other tissues in left-sided breast cancer patients with poor cardiac anatomy^17,20,40–42^. Mean heart dosage is a good prognosticator to monitor the heart sequelae^43^. In the present study, mean dosage to the heart was 4.9 Gy in the entire cohort, and the median V25 Gy to the heart was 5.3% and 3.1% in Tomotherapy and IMRT respectively (*P* = 0.04) Such difference did not affect OS, DMFS or CSS as shown in Table 2. We will continue to follow this cohort.

The present study has the inter-observer variability, since the physicians involved in the toxicity scoring were not blinded; besides, helical Tomotherapy rather than TomoDirect was utilized due to institutional facility restrictions. Most of all, obvious limitation is its retrospective nature.

## Conclusions

In the setting of IMRT-SIB, Tomotherapy improved acute skin toxicity compared with forward IMRT-SIB. Chronic skin complications reached 1.9%. Both forward IMRT-SIB and inverse IMRT-SIB via Tomotherapy resulted in good 5-year and 7-year survival. Longer follow-up is intended.

## Data Availability

The data used to support the findings of this study are included within the article.
